# Supplementary material for: Allosteric activation of cell wall synthesis during bacterial growth
Source: Nat Commun. 2023 Jun 10;14:3439. doi: 10.1038/s41467-023-39037-9 (PMC10257715; doi:10.1038/s41467-023-39037-9)
Supplement: Supplementary file 1 — Supplementary Information [file 41467_2023_39037_MOESM1_ESM.pdf]

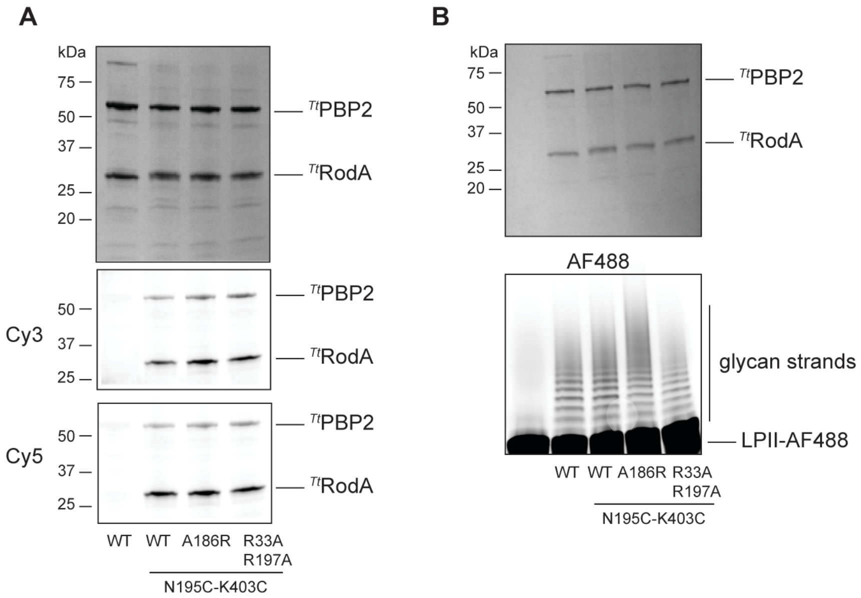

Supplementary Figure 1. Biochemical validation of ^{Tt}RodA-PBP2 smFRET imaging constructs. A. Gels of fluorophore-labeled double-cysteine imaging mutants and mock-labeled ^{Tt}WT control show specific incorporation of Cy3 and Cy5 fluorophores in RodA and PBP2 (pMS239, pSI7, pSI12, pSI13). Top: coomassie-stained control; middle: Cy3 signal; bottom: Cy5 signal. Images are representative of n=2 independent experiments. B. Imaging mutants retain activity after labeling (same constructs as A). Top: coomassie-stained control; bottom: Cy2 signal, showing polymerization of AF488-labeled lipid II. Images are representative of n=2 independent experiments.

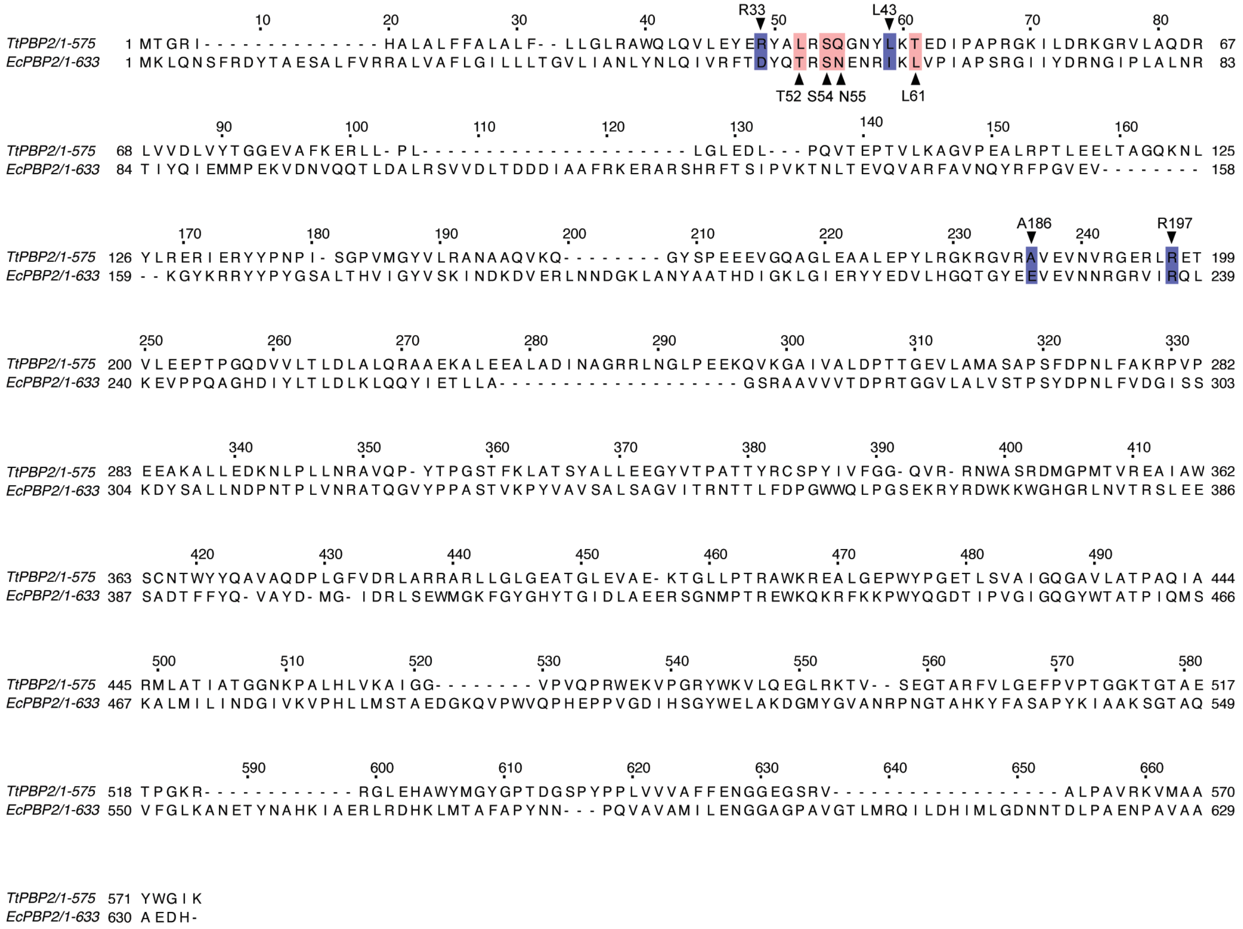

Supplementary Figure 2. Sequence alignment (Jalview) between *T. thermophilus* PBP2 and *E. coli* PBP2 showing the positions of hinge and interface mutants in blue (^{Tt}PBP2) and in pink (^{Ec}PBP2).

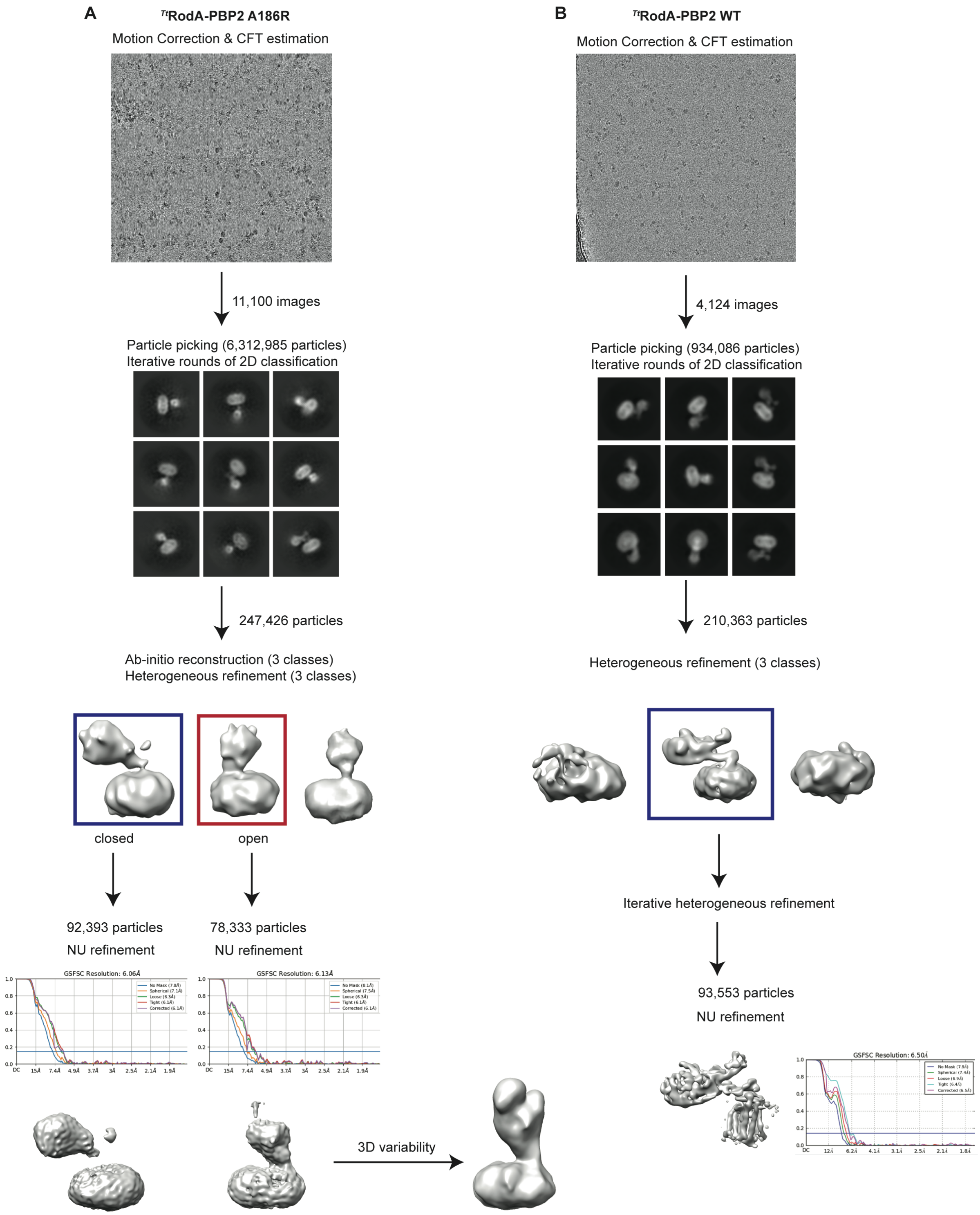

Supplementary Figure 3. Cryo-EM processing workflow for ^{Tt}RodA-PBP2 WT (A) and A186R mutant (B) samples.

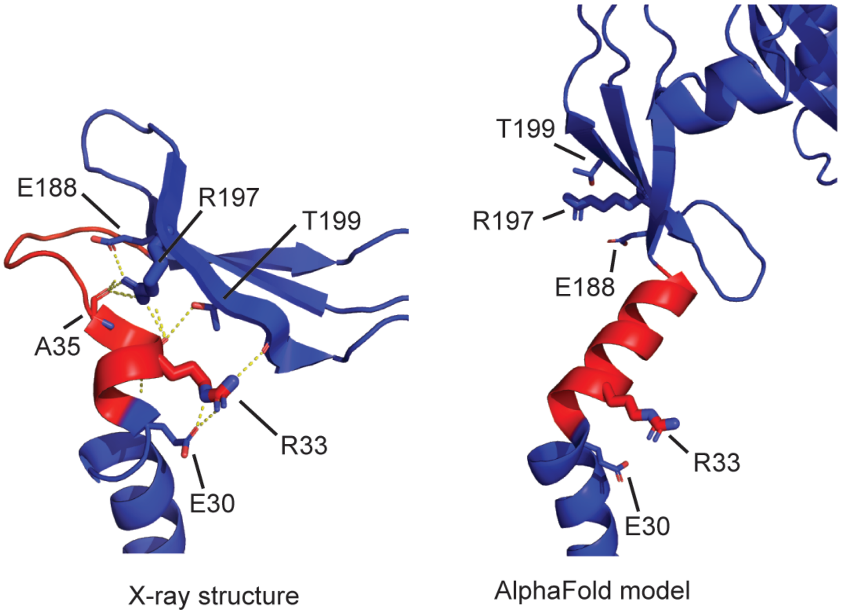

Supplementary Figure 4. Close-up view of the hinge region as in Fig. 2D., showing that the tight hydrogen-bond network mediated by R33 and R197 is lost in the open state.

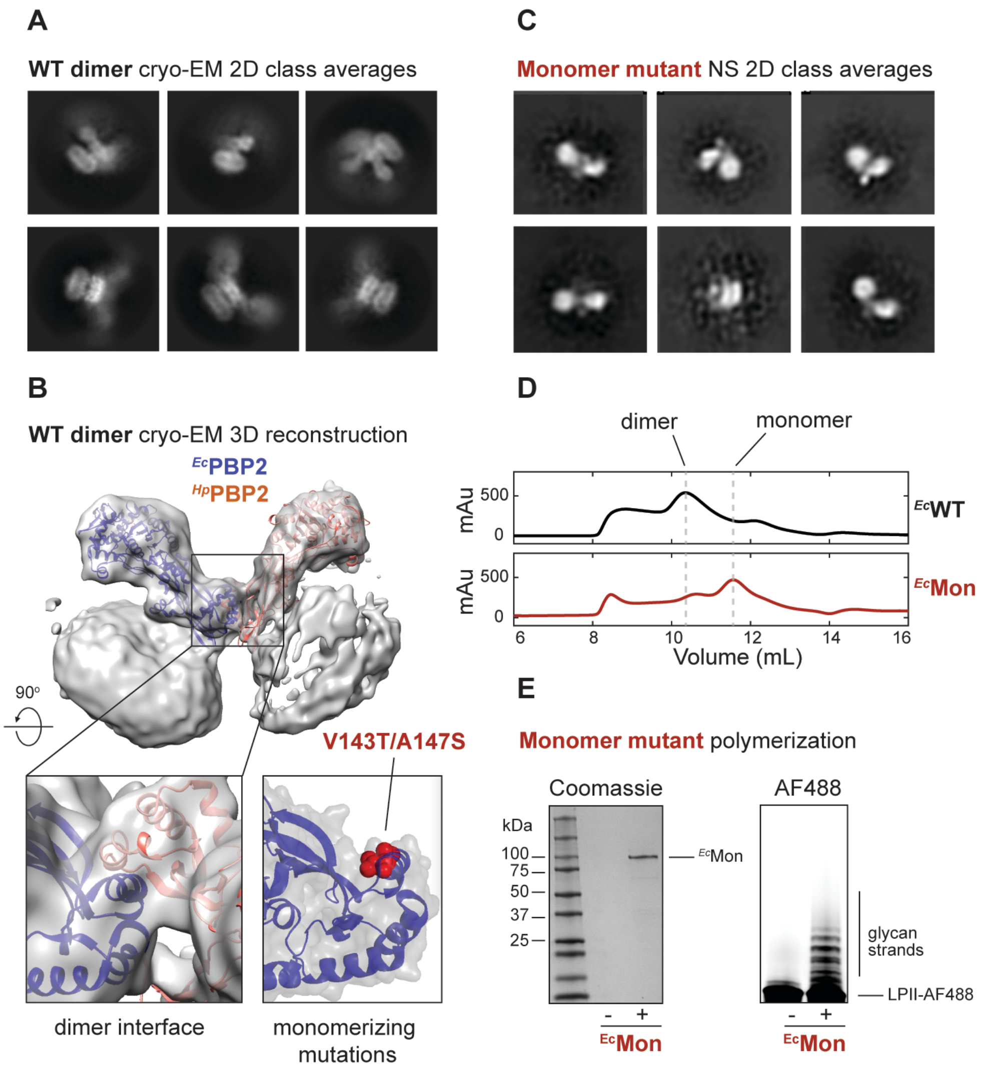

Supplementary Figure 5. Designing a monomeric ^{Ec}RodA-PBP2 construct.
A. Representative cryo-EM 2D class averages of ^{Ec}RodA-PBP2^{WT} fusion construct (pSS50) show that the protein forms 2 types of dimers (1:1 protein:micelle and 2:1 protein:micelle). Each class contains 2-2.5k particles. B. 3D reconstruction of the main population of 1:1 protein:micelle dimeric particles. The conformation of the pedestal domain of PBP2 appeared distinct in the two protomers: either with the anchor and head domains closed (blue), similar to X-ray structure of ecto-^{Ec}PBP2 (PDB: 6g9f), or open (orange), similar to the MreC-bound state of ecto-^{Hp}PBP2 (PDB: 5lp5). To resolve the dimer interface, ^{Ec}PBP2 and ^{Hp}PBP2 crystal structures were fit into the cryo-EM map, assuming a symmetric dimer (bottom left). Mutations (red spheres) were designed to disrupt dimerization by increasing the hydrophilicity of the dimerization helix (right). C. Representative negative-stain electron microscopy 2D class averages show that ^{Ec}RodA-PBP2$^{V143T\text{-}A147S}$ mutant is monomeric (^{Ec}Mon, pSI108). Each class contains ~500 particles. D. V143T-A147S mutations shift the size-exclusion elution profile of ^{Ec}RodA-PBP2. E. Monomeric mutant retains polymerization activity. Left: coomassie-stained control; right: Cy2 excitation, showing polymerization of AF488-labeled lipid II. Images are representative of n=2 independent experiments.

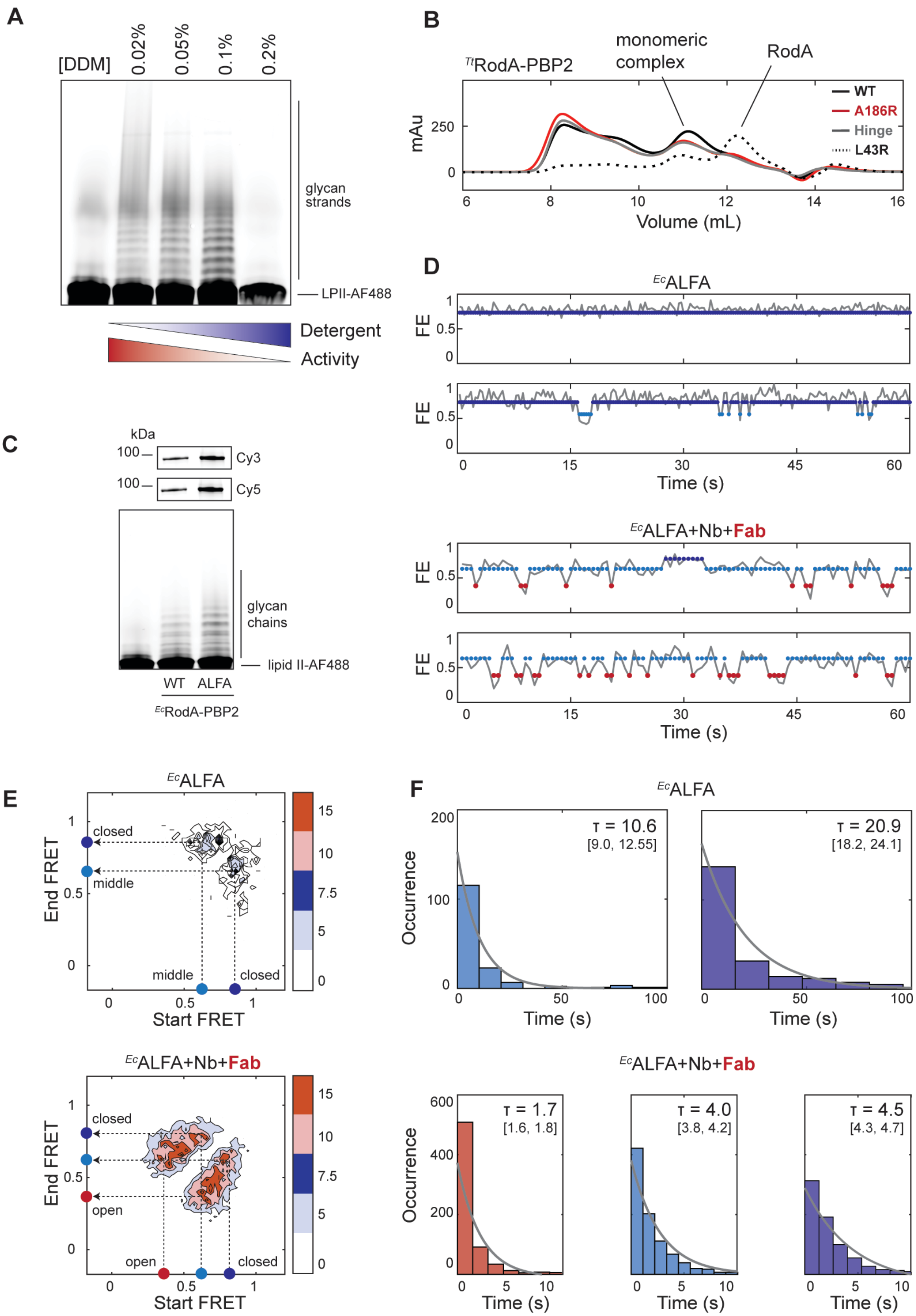
A
[DDM]
0.02%
0.05%
0.1%
0.2%
glycan strands
LPII-AF488
Detergent
Activity
B
TtRodA-PBP2
monomeric complex
RodA
WT
A186R
Hinge
L43R
mAu
Volume (mL)
C
kDa
100
Cy3
Cy5
glycan chains
lipid II-AF488
WT
ALFA
EcRodA-PBP2
D
EcALFA
FE
Time (s)
EcALFA+Nb+Fab
E
closed
middle
open
End FRET
Start FRET
F
τ = 10.6
[9.0, 12.55]
τ = 20.9
[18.2, 24.1]
τ = 1.7
[1.6, 1.8]
τ = 4.0
[3.8, 4.2]
τ = 4.5
[4.3, 4.7]
Occurrence

Supplementary Figure 6. Biochemical validation of constructs used in polymerization assays. A. Detergent concentration dramatically affects polymerization activity. Polymerization activity of ^{Ec}RodA-PBP2^{Mon} (pSI126) assayed under a range of DDM concentrations above CMC (0.02-0.2%). AF488-labeled glycan chains were visualized with Cy2 excitation. Images are representative of n=2 independent experiments. B. Size-exclusion elution (SEC) profiles of *T. thermophilus* RodA-PBP2 WT and mutants (pSI7, pSI12, pSI13, pMS292). All PBP2 mutants retain WT fold and form a stable complex with RodA except for L43R, which shows considerable dissociation into ^{Tt}RodA and ^{Tt}PBP2. C. ^{Ec}RodA-PBP2^{ALFA} retains activity after labeling. Top: Cy3 and Cy5 signals, showing fluorophore labeling; bottom: Cy2 signal, showing polymerization of AF488-labeled lipid II. Images are representative of n=2 independent experiments. D. Representative trajectories of ^{Ec}ALFA with 1:1 Nb:Fab complex from Fig. 4C. Mean values of states are marked. E. Transition density plots, normalized to total observation time, show that dynamic exchange into the low-FRET state is dramatically increased upon addition of the Fab-Nb complex. F. Dwell time histograms and exponential fits for the low-FRET (red), middle-FRET (light blue) and high-FRET (blue) states for ^{Ec}ALFA with or without Nb-Fab. Mean dwell times alongside 95% confidence intervals for all populations are indicated on the plots.

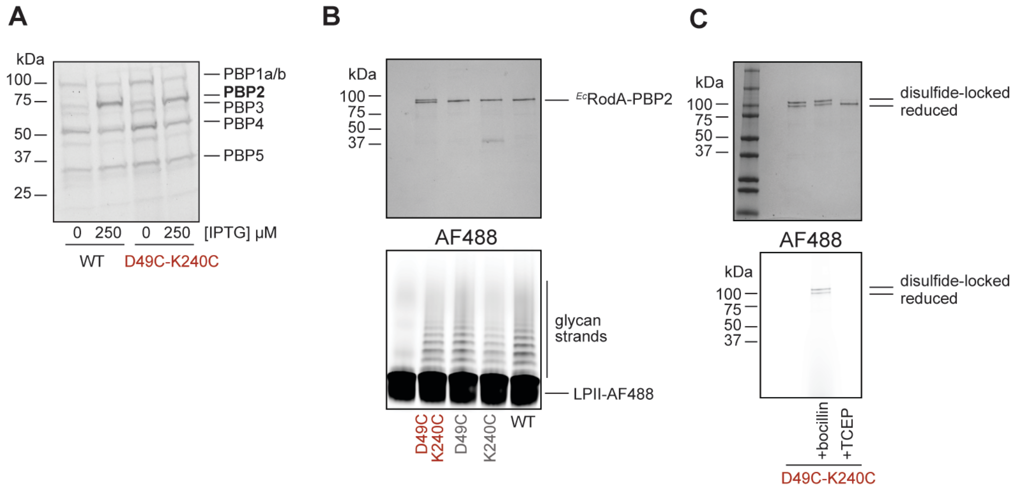

Supplementary Figure 7. Disulfide lock does not impair protein expression or fold.

A. ^{Ec}RodA-PBP2$^{D49C\text{-}K240C}$ mutant expresses at 0.5-1x of ^{Ec}RodA-PBP2^{WT}. Bocillin labeling was used to visualize and quantify PBP2 expression levels upon induction with IPTG in complementation strains FB38/pHC857 and FB38/pSI47 (as in Fig. 5C). Image shown is representative of n=2 independent experiments. B. Polymerization assay, showing that introduction of cysteine mutations does not impair activity. Top: coomassie control. Bottom: AF488 excitation, showing polymerization of AF488-labeled lipid II. Images are representative of n=2 independent experiments. C. ^{Ec}PBP2$^{D49C\text{-}K240C}$ (pSI128) forms a disulfide with ~50% efficiency. Top: SDS-PAGE shows that ^{Ec}D49C-K240C sample contains a second band, in ^{Ec}Mon (compare with Fig. S3E), which disappears upon treatment with TCEP. The top and bottom bands were assigned to the disulfide-locked and reduced species, respectively. The efficiency of disulfide formation was quantified from the relative intensities of the two bands. Bottom: bocillin labeling visualized with Cy2 excitation. Images are representative of n=3 independent experiments.

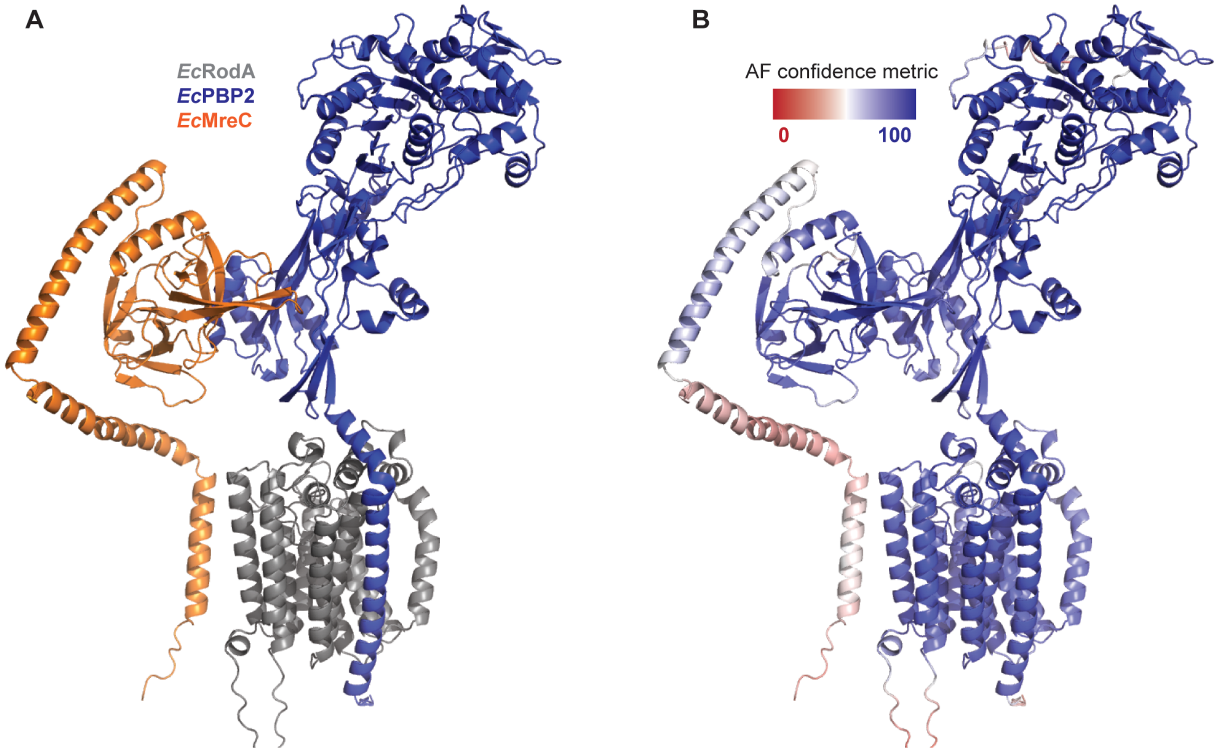

Supplementary Figure 8. AlphaFold model of the ^{Ec}RodA-PBP2-MreC complex.
A. AlphaFold model of the complex between ^{Ec}RodA (grey), ^{Ec}PBP2 (blue) and ^{Ec}MreC monomer (orange). The model predicts that MreC binds the open configuration of PBP2 through contacts in the pedestal domain. B. The same complex as in A., colored by the per residue confidence metric (pLDDT). The binding interface between PBP2 and MreC is predicted with high confidence.

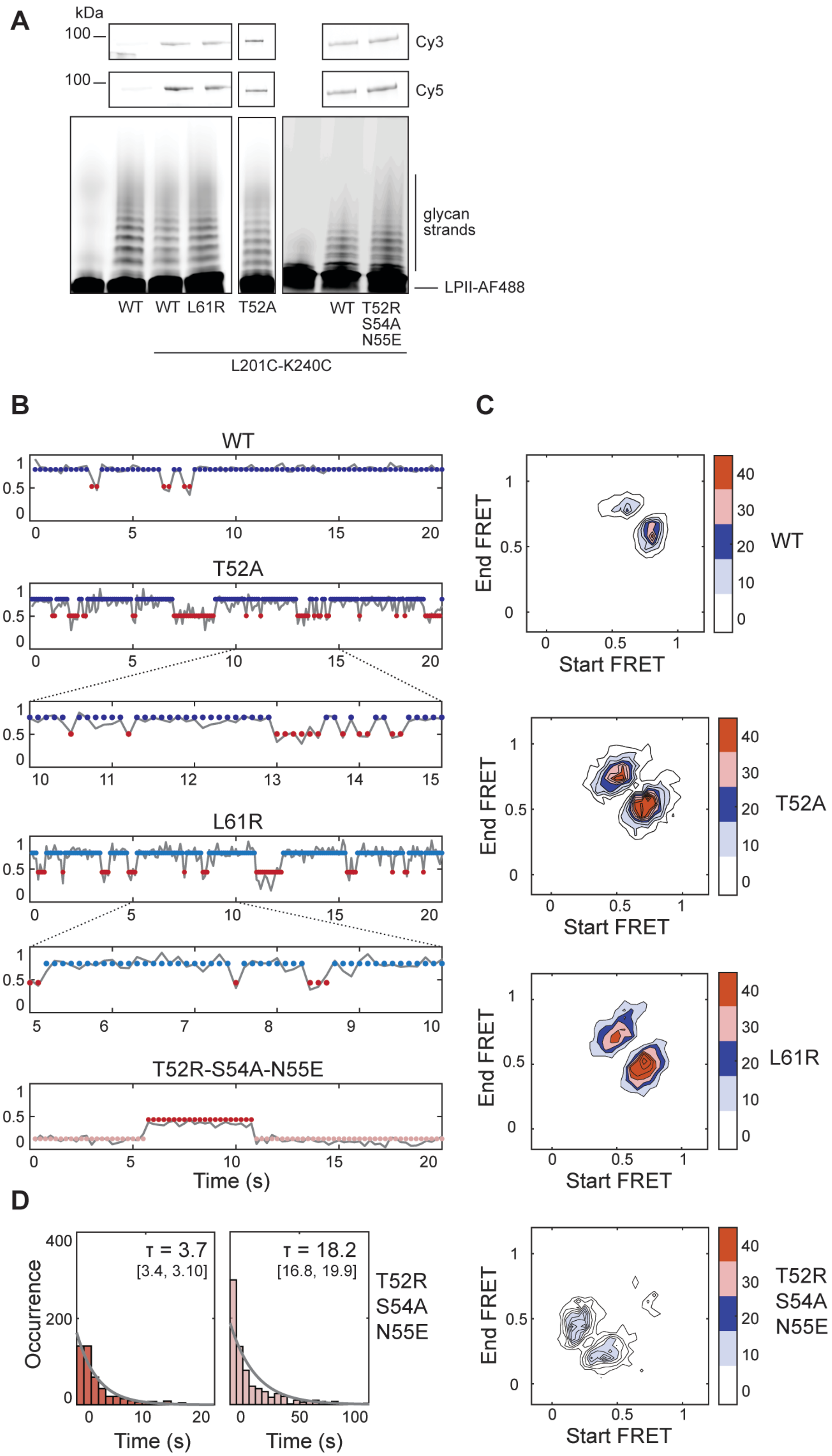

A
kDa
100
100
Cy3
Cy5
glycan strands
LPII-AF488
WT
WT
L61R
T52A
WT
T52R S54A N55E
L201C-K240C
B
WT
T52A
L61R
T52R-S54A-N55E
FE
Time (s)
C
End FRET
Start FRET
WT
T52A
L61R
T52R S54A N55E
D
Occurrence
τ = 3.7
[3.4, 3.10]
τ = 18.2
[16.8, 19.9]
T52R S54A N55E
Time (s)

Supplementary Figure 9. Biochemical validation of ^{Ec}RodA-PBP2 smFRET imaging constructs. A. smFRET imaging constructs are specifically labeled with Cy3 and Cy5 fluorophores and retain activity after labeling. Top: Labeling gels of the double-cysteine imaging mutants and the mock-labeled ^{Ec}RodA-PBP2^{WT} control (pSI131, pSI126, pSI127, pSI149, pSI152). Bottom: Lipid II-AF488 polymerization assay with the same constructs. Images are representative of n=2 independent experiments. B. Representative trajectories of smFRET constructs from Fig. 6B. ^{Ec}RodA-PBP2^{Mon} trajectory is reproduced from Fig. 3 for comparison. The insets show expansions of ^{Ec}T52A and ^{Ec}L61R trajectories to highlight individual transition events. Note that these mutants were imaged at a higher frame rate than the rest of the constructs (10 s^{-1} vs 4 s^{-1}) to capture their faster dynamics. C. Transition plot analysis shows a comparison of transition frequency between dynamic mutants and ^{Ec}Mon (reproduced from Fig. 3C), normalized to total observation time. D. Dwell time histograms and exponential fits for the lowest-FRET (pink), and low-FRET (red) states for ^{Ec}RodA-PBP2$^{T52R\text{-}S54A\text{-}N55E}$. Mean dwell times alongside 95% confidence intervals for all populations are indicated on the plots.

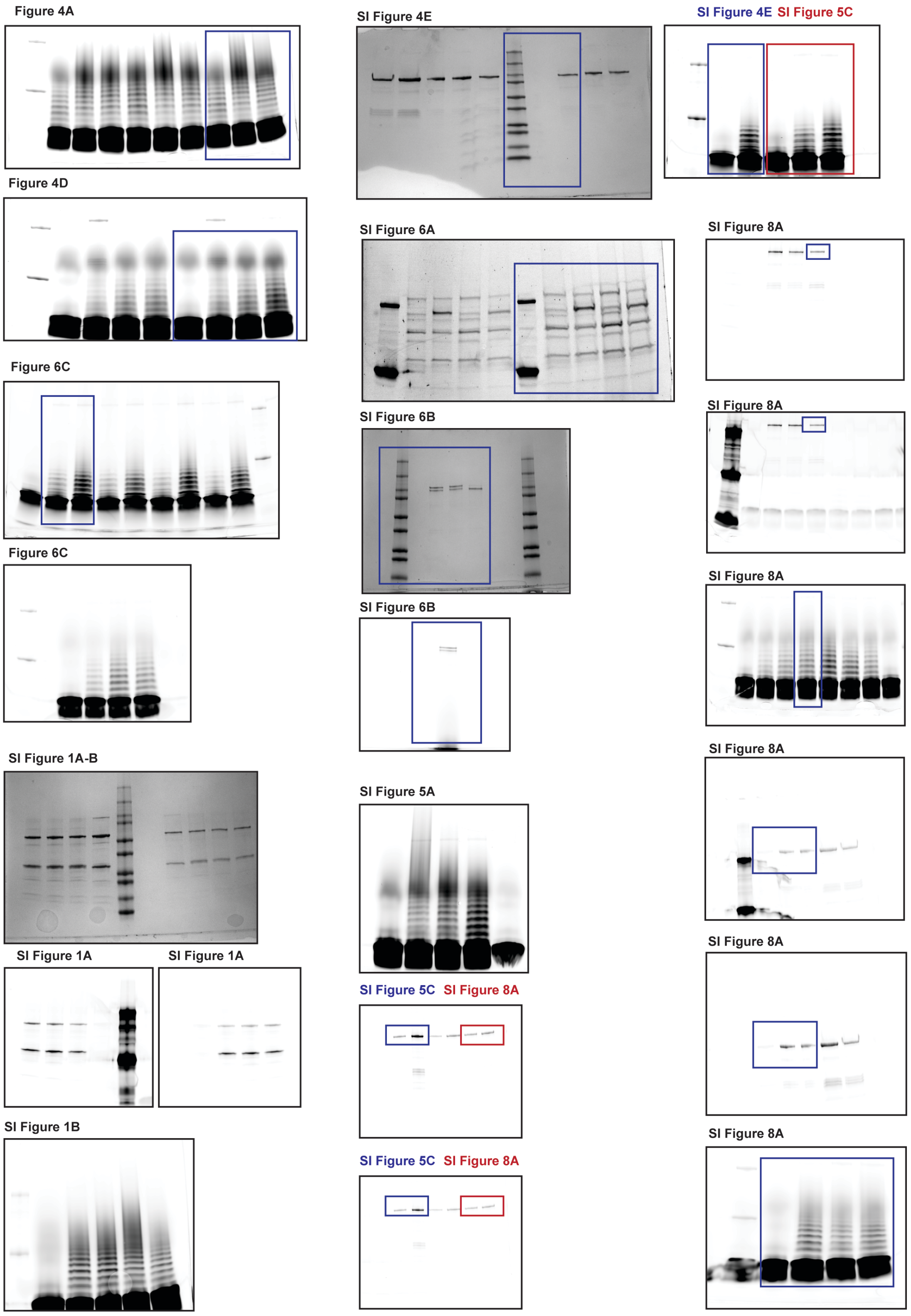

Supplementary Figure 10. Uncropped gels organized by figure. The regions shown in figures are marked with rectangles.

Supplementary Table 1. smFRET trajectory parameters

	Construct	Trajectories	Observations	Transitions	PDF fits (means)
^{Tt}RodA-PBP2	WT	222	15,984	124*	0.83
	R33A-R197A	436	33,665	540	0.49<br>0.8
	A186R	415	38,978	779	0.45<br>0.75
^{Ec}RodA-PBP2	WT	270	59,064	2045	0.52<br>0.78
	ALFA	94	17,423	400	0.60<br>0.87
	ALFA + Nb-Fab	122	30,341	4196	0.38<br>0.63<br>0.77
	T52A	173	66,054	2684	0.5<br>0.78
	L61R	100	35,745	1886	0.42<br>0.67<br>0.90
	T52R-S54A-N55E	195	45,262	1048	0.16<br>0.45

*Note that virtually none of ^{Tt}WT "transitions" are bona fide, i.e., they are an artifact of fitting the data to a two-state model and correspond to exchange within the high-FRET state, rather than to true cross-state exchange.

Supplementary Table 2. Cryo-EM sample preparation and data collection

	^{Tt}RodA-PBP2^{WT}	^{Tt}RodA-PBP2^{A186R}	^{Ec}RodA-PBP2^{WT}
Grid type	C-flat holey carbon	C-flat holey carbon	UltrAuFoil holey gold
Protein concentration	3.5 mg/mL	3 mg/mL	3 mg/mL
Blotting parameters	7 s blot time<br>15 blot force	7 s blot time<br>15 blot force	7 s blot time<br>15 blot force
Microscope	Titan Krios	Titan Krios	Titan Krios
Pixel size (Å)	0.825	0.825	0.825
Voltage (kV)	300	300	300
Detector	K3	K3	K3
Total exposure (e-/Å)	64	58	59
Defocus range (μm)	−1 to −2.5	−1.2 to −2.5	−1.2 to −2.5
Number of frames per micrograph	50	54	57
Exposure time (s)	3	1.4	2
Final particles	93,553	78,333 (open)<br>92,393 (closed)	78,031 (1:1 protein: micelle dimer)
Symmetry	C1	C1	C1
Nominal resolution (Å)	6.5	6.1 (closed)<br>NA (open)	9

Supplementary Table 3: bacterial strains used for *in vivo* assays

Strain	Genotype	Source
MG1655	*rph1 ilvG rfb-50*	Guyer et al. 1981
MG1655/pSI56	*rph1 ilvG rfb-50* / P_{tac}:*pbpA-rodA*	This study
MG1655/pSI57	*rph1 ilvG rfb-50* / P_{tac}:*pbpA(D49C-K240C)-rodA*	This study
MG1655/pSI58	*rph1 ilvG rfb-50* / P_{tac}:*pbpA(D49C)-rodA*	This study
MG1655/pSI59	*rph1 ilvG rfb-50* / P_{tac}:*pbpA(K240C)-rodA*	This study
MG1655/pSI63	*rph1 ilvG rfb-50* / P_{tac}:*pbpA(S330A)-rodA*	This study
MG1655/pHC857	*rph1 ilvG rfb-50* / P_{lac}:*pbpA-rodA*	This study
MG1655/pHC858	*rph1 ilvG rfb-50* / P_{lac}:*pbpA(S330A)-rodA*	This study
MG1655/pSI47	*rph1 ilvG rfb-50* / P_{lac}:*pbpA(D49C-K240C)-rodA*	This study
MG1655/pSI48	*rph1 ilvG rfb-50* / P_{lac}:*pbpA(D49C)-rodA*	This study
MG1655/pSI49	*ph1 ilvG rfb-50* / P_{lac}:*pbpA(K240C)-rodA*	This study
MG1655/pSI154	*rph1 ilvG rfb-50* / P_{lac}:*pbpA(T52A)-rodA*	This study
MG1655/pSI157	*rph1 ilvG rfb-50* / P_{lac}:*pbpA(T52R-S54A-N55E)-rodA*	This study
MG1655/pRY47	*rph1 ilvG rfb-50* / P_{lac}: empty	This study
FB38	*rph1 ilvG rfb-50 ΔlacIZYA::frt mrdAB::aph*	Bendezu & de Boer, 2008
FB38/pHC857	*rph1 ilvG rfb-50 ΔlacIZYA::frt mrdAB::aph* / P_{lac}:*pbpA-rodA*	P1(FB38) x MG1655/pHC857
FB38/pHC858	*rph1 ilvG rfb-50 ΔlacIZYA::frt mrdAB::aph* / P_{lac}:*pbpA(S330A)-rodA*	P1(FB38) x MG1655/pHC858
FB38/pSI47	*rph1 ilvG rfb-50 ΔlacIZYA::frt mrdAB::aph* / P_{lac}:*pbpA(D49C-K240C)-rodA*	P1(FB38) x MG1655/pSI47
FB38/pSI48	*rph1 ilvG rfb-50 ΔlacIZYA::frt mrdAB::aph* / P_{lac}:*pbpA(D49C)-rodA*	P1(FB38) x MG1655/pSI48
FB38/pSI49	*rph1 ilvG rfb-50 ΔlacIZYA::frt mrdAB::aph* / P_{lac}:*pbpA(K240C)-rodA*	P1(FB38) x MG1655/pSI49

PR5	*rph1 ilvG rfb-50 mreC(R292H) yrdE-kan*	Rohs et al. 2018
PR5/pHC857	*rph1 ilvG rfb-50 mreC(R292H) yrdE-kan* / P_{lac}:*pbpA-rodA*	P1(PR5) x MG1655/pHC857
PR5/pSI154	*rph1 ilvG rfb-50 mreC(R292H) yrdE-kan* / P_{lac}:*pbpA(T52A)-rodA*	P1(PR5) x MG1655/pSI154
PR5/pSI157	*rph1 ilvG rfb-50 mreC(R292H) yrdE-kan* / P_{lac}:*pbpA(T52R-S54A-N55E)-rodA*	P1(PR5) x MG1655/pSI157
PR5/pRY47	*rph1 ilvG rfb-50 mreC(R292H) yrdE-kan* / P_{lac}:*empty*	P1(PR5) x MG1655/pRY47

Supplementary Table 4: plasmids used for *in vivo* assays

**Plasmid**	**Genotype**	**ori**	**Source**
pHC857	*cat lacI^q^* P_{lac}:*pbpA-rodA*	pBR/colE1	Cho et al. 2014
pHC858	*cat lacI^q^* P_{lac}:*pbpA(S330A)-rodA*	pBR/colE1	Cho et al. 2014
pSI47	*cat lacI^q^* P_{lac}:*pbpA(D49C-K240C)-rodA*	pBR/colE1	This study
pSI48	*cat lacI^q^* P_{lac}:*pbpA(D49C)-rodA*	pBR/colE1	This study
pSI49	*cat lacI^q^* P_{lac}:*pbpA(K240C)-rodA*	pBR/colE1	This study
pHC800	*cat lacI^q^* P_{tac}	pBR/colE1	Cho et al. 2014
pSI56	*cat lacI^q^* P_{tac}:*pbpA-rodA*	pBR/colE1	This study
pSI57	*cat lacI^q^* P_{tac}:*pbpA(D49C-K240C)-rodA*	pBR/colE1	This study
pSI58	*cat lacI^q^* P_{tac}:*pbpA(D49C)-rodA*	pBR/colE1	This study
pSI59	*cat lacI^q^* P_{tac}:*pbpA(K240C)-rodA*	pBR/colE1	This study
pSI63	*cat lacI^q^* P_{tac}:*pbpA(S330A)-rodA*	pBR/colE1	This study
pSI154	*cat lacI^q^* P_{lac}:*pbpA(T52A)-rodA*	pBR/colE1	This study
pSI157	*cat lacI^q^* P_{lac}:*pbpA(T52R-S54A-N55E)-rodA*	pBR/colE1	This study
pRY47	*cat lacI^q^* P_{lac}: empty	pBR/colE1	Cho et al. 2014

Supplementary Table 5: plasmids used for *in vitro* assays

Plasmid	Genotype	Source
pMS239	*ColA-T7:TtPBP2-3C-PrtC; P_{T7}:His6-SUMO-Flag-3C-TtRodA*	Sjodt et al. 2020
pMS292	*ColA-T7:TtPBP2(L43R)-3C-PrtC; P_{T7}:His6-SUMO-Flag-3C-TtRodA*	
pMS293	*ColA-T7:TtPBP2(A186R)-3C-PrtC; P_{T7}:His6-SUMO-Flag-3C-TtRodA*	Sjodt et al. 2020
pSI7	*ColA-T7:TtPBP2(K403C)-3C-PrtC; P_{T7}:His6-SUMO-Flag-Avitag-TtRodA(N195C)*	This study
pSI12	*ColA-T7:TtPBP2(A186R-K403C)-3C-PrtC; P_{T7}:His6-SUMO-Flag-Avitag-TtRodA(N195C)*	This study
pSI13	*ColA-T7:TtPBP2(R33A-R197A-K403C)-3C-PrtC; P_{T7}:His6-SUMO-Flag-Avitag-TtRodA(N195C)*	This study
pSS50	*pAM205-T7:His6-SUMO-Flag-3C-EcPBP2-GGGSx3-EcRodA*	Rohs et al. 2018
pSI108	*T7:His6-SUMO-Flag-3C-EcPBP2(V143T-A147S)-GGGSx3-EcRodA*	This study
pSI126	*T7:His6-SUMO-Flag-3C-EcPBP2(V143T-A147S-R425C)-GGGSx3-EcRodA(C133A-L201C)*	This study
pSI147	*T7:His6-SUMO-Flag-3C-EcPBP2(V143T-A147S-[I182-ALFA tag-A201] R425C)-GGGSx3-EcRodA(C133A-L201C)*	This study
pSI127	*T7:His6-SUMO-Flag-3C-EcPBP2(L61R-V143T-A147S-R425C)-GGGSx3-EcRodA(C133A-L201C)*	This study
pSI128	*T7:His6-SUMO-Flag-3C-EcPBP2(D49C-K240C-V143T-A147S)-GGGSx3-EcRodA(C133A)*	This study
pSI129	*T7:His6-SUMO-Flag-3C-EcPBP2(D49C-V143T-A147S)-GGGSx3-EcRodA(C133A)*	This study
pSI130	*T7:His6-SUMO-Flag-3C-EcPBP2(K240C-V143T-A147S)-GGGSx3-EcRodA(C133A)*	This study
pSI131	*T7:His6-SUMO-Flag-3C-EcPBP2(V143T-A147S)-GGGSx3-EcRodA(C133A)*	This study

pSI149	*T7:His6-SUMO-Flag-3C-EcPBP2(T52A-V143T-A147S-R425C)-GGGSx3-EcRodA(C133A-L201C)*	This study
pSI152	*T7:His6-SUMO-Flag-3C-EcPBP2(T52R-S54A-N55E-V143T-A147S-R425C)-GGGSx3-EcRodA(C133A-L201C)*	This study
pSI145	*pET26b-T7:PelB-NbALFA-6xHis*	This study, nanobody sequence from Götzke & Kilisch et al. 2019
pSI146	*pET26b-T7:PelB-NbALFA(Q116K-Q119P)-6xHis*	This study
pMAS478	*pTarget:* heavy chain	This study, Fab sequence from Bloch et al. 2021
pMAS481	*pD2610-v5:* light chain	This study, Fab sequence from Bloch et al. 2021

Supplementary Table 6: primers used for Gibson assembly

Primer name	Primer sequence
N195C.fwd<br>N195C.rev	5'-ccaactgccgtctggccttgtctgaaaccatatcaacg-3'<br>5-cgttgatatggtttcagacaaggccagacggcagttgg-3'
K403C.fwd<br>K403C.rev	5'- ggaagttgcagagtgcaccgggcttcttcccactcgc-3'<br>5-gcgagtgggaagaagcccggtgcactctgcaacttcc-3'
A186R.fwd<br>A186R.rev	5'- cgcggggtgcgtcgagtagaggtcaatgtg-3'<br>5'- gcacattgacctctactcgacgcaccccgcg-3'
R33A.fwd<br>R33A.rev	5'-caggtgctggaatatgaggcttatgcgcttcgc-3'<br>5'-gcgaagcgcataagcctcatattccagcacctg-3'
R197A.fwd<br>R197C.rev	5'-gcgcggtgaacgcttagccgaaacagttttagagg-3'<br>5'-cctctaaaactgtttcggctaagcgttcaccgcgc-3'
V143T-A147S.fwd<br>V143T-A147S.rev	5'-gaccgaagtacaaacagctcgcttttccgtcaatcag -3'<br>5'-ctgattgacggaaaagcgagctgtttgtacttcggtc -3'
R425C.fwd<br>R425C.rev	5'-ctggcggaagaatgctccggcaacatgc -3'<br>5'-catgttgccggagcattcttccgccagg -3'
C133A.fwd<br>C133A.rev	5'-caaccgcgacgttgccccgccatcgtt-3'<br>5'-aacgatggcggggcaacgtcgcggttg-3'

L201C.fwd<br>L201C.rev	5’-ctgtggttcttctgcatgcatgattaccagcgc-3’<br>5’-gcgctggtaatcatgcatgcagaagaaccacag-3’
L61R.fwd<br>L61R.rev	5’-gaaaaccgcattaagcgtgtgcctatcgcg-3’<br>5’-gcgcgataggcacacgcttaatgcggttttc-3’
T52A.fwd<br>T52A.rev	5’-ccgactaccaggcccgctctaatgaaaac-3’<br>5’-gttttcattagagcgggcctggtagtcgg-3’
T52R-S54A-N55E.fwd<br>T52R-S54A-N55E.rev	5’-cgctttaccgactaccagcgccgcgctgaggaaaaccgcattaagc-3’<br>5’-gcttaatgcggttttcctcagcgcggcgctggtagtcggtaaagcg-3’
D49C.fwd<br>D49C.rev	5’-gttcgctttacctgctaccagacccgc-3’<br>5’-gcgggtctggtagcaggtaaagcgaac-3’
K240C.fwd<br>K240C.rev	5’-gttattcgccagttatgtgaagtaccaccgcaagc-3’<br>5’-gcttgcggtggtacttcacataactggcgaataac-3’
