# Supplementary material for: Allosteric activation of cell wall synthesis during bacterial growth
Source: Nat Commun. 2023 Jun 10;14:3439. doi: 10.1038/s41467-023-39037-9 (PMC10257715; doi:10.1038/s41467-023-39037-9)
Supplement: Supplementary file 3 — Reporting Summary [file 41467_2023_39037_MOESM3_ESM.pdf]

nature portfolio

Corresponding author(s): Joseph J. Loparo, Andrew C. Kruse

Last updated by author(s): 05/03/2023

Reporting Summary

Nature Portfolio wishes to improve the reproducibility of the work that we publish. This form provides structure for consistency and transparency in reporting. For further information on Nature Portfolio policies, see our Editorial Policies and the Editorial Policy Checklist.

Statistics

For all statistical analyses, confirm that the following items are present in the figure legend, table legend, main text, or Methods section.

n/a	Confirmed	
☐	☒	The exact sample size (*n*) for each experimental group/condition, given as a discrete number and unit of measurement
☐	☒	A statement on whether measurements were taken from distinct samples or whether the same sample was measured repeatedly
☐	☒	The statistical test(s) used AND whether they are one- or two-sided<br>*Only common tests should be described solely by name; describe more complex techniques in the Methods section.*
☒	☐	A description of all covariates tested
☐	☒	A description of any assumptions or corrections, such as tests of normality and adjustment for multiple comparisons
☐	☒	A full description of the statistical parameters including central tendency (e.g. means) or other basic estimates (e.g. regression coefficient) AND variation (e.g. standard deviation) or associated estimates of uncertainty (e.g. confidence intervals)
☐	☒	For null hypothesis testing, the test statistic (e.g. F, t, r) with confidence intervals, effect sizes, degrees of freedom and P value noted<br>*Give P values as exact values whenever suitable.*
☐	☒	For Bayesian analysis, information on the choice of priors and Markov chain Monte Carlo settings
☒	☐	For hierarchical and complex designs, identification of the appropriate level for tests and full reporting of outcomes
☒	☐	Estimates of effect sizes (e.g. Cohen's *d*, Pearson's *r*), indicating how they were calculated

Our web collection on statistics for biologists contains articles on many of the points above.

Software and code

Policy information about availability of computer code

Data collection	Fluorescence images were acquired using a microscope controlled by Hamamatsu HCImage live version 4.4.0.1 and Labview version 15.0f2. Cryo-electron micrographs were collected on Titan Krios operating at 300 kV and equipped with a K3 camera. Phase contrast microscopy was performed on a Nikon Ti2-E Inverted Motorized Microscope equipped with a Plan Apo 100x/1.45 Oil Ph3 objective and a 4.2bi Back Illuminated Cooled sCMOS camera (PCO). Images were acquired using Nikon Elements 5.2 Acquisition Software.<br>Details are provided in the Methods.
Data analysis	Fluorescence imaging analysis was performed in MATLAB using previously described custom written codes (Graham et a, Mol Cell. 2016, 61, 850-858); ebFRET (van de Meent et al, Biophys J. 2014, 106, 1327-1337) and ImageJ2 version 2.9.0/1.53t. Cryo-EM data processing was carried out in CryoSPARC v2 or v3 (Punjani et al, Nat Methods. 2017, 14, 290-296) and cryo-EM maps were visualized in Chimera version 1.15. Phase contrast images were processed to quantify aspect ratio using MicrobeJ in ImageJ2 (Ducret et al, Nature Microbiology. 2016, 1, 16077).<br>Details are provided in the Methods.

For manuscripts utilizing custom algorithms or software that are central to the research but not yet described in published literature, software must be made available to editors and reviewers. We strongly encourage code deposition in a community repository (e.g. GitHub). See the Nature Portfolio guidelines for submitting code & software for further information.

Data

Policy information about availability of data

All manuscripts must include a data availability statement. This statement should provide the following information, where applicable:

- Accession codes, unique identifiers, or web links for publicly available datasets
- A description of any restrictions on data availability
- For clinical datasets or third party data, please ensure that the statement adheres to our policy

Source data are provided with this paper. Uncropped SDS-PAGE gels and western blots from all the figures are provided as Supplementary Figure 10. Supporting data are publicly available on Zenodo (https://doi.org/10.5281/zenodo.7884683).

Human research participants

Policy information about studies involving human research participants and Sex and Gender in Research.

Reporting on sex and gender	N/A
Population characteristics	N/A
Recruitment	N/A
Ethics oversight	N/A

Note that full information on the approval of the study protocol must also be provided in the manuscript.

Field-specific reporting

Please select the one below that is the best fit for your research. If you are not sure, read the appropriate sections before making your selection.

☒ Life sciences ☐ Behavioural & social sciences ☐ Ecological, evolutionary & environmental sciences

For a reference copy of the document with all sections, see nature.com/documents/nr-reporting-summary-flat.pdf

Life sciences study design

All studies must disclose on these points even when the disclosure is negative.

Sample size	Sample sizes for all datasets are listed in the figure captions, the Methods section or in the Supplementary material. For in vitro polymerization assays, 3-4 technical repeats per biological replicate were used, and 2-3 biological replicates were performed for each experiment, to account for both technical and biological variability. For smFRET imaging, each dataset includes 150-400 trajectories collected from a minimum of two independent experiments, which provides sufficient sampling to fit population and dynamic parameters with confidence.
Data exclusions	For fluorescence image analysis, individual molecules were automatically excluded if they violated a set of criteria (ellipticity, proximity to other molecules analyzed, multiple labels etc as outlined in the Methods).
Replication	All results presented in this study have been replicated in at least two independent biological experiments (and where applicable 3-4 technical replicates each).
Randomization	This is not a clinical study that includes human subjects. Thus, randomization was not relevant to this study.
Blinding	Image analysis for both smFRET and in vivo morphology experiments was carried in an automated fashion, therefore blinding was not relevant to this study.

Reporting for specific materials, systems and methods

We require information from authors about some types of materials, experimental systems and methods used in many studies. Here, indicate whether each material, system or method listed is relevant to your study. If you are not sure if a list item applies to your research, read the appropriate section before selecting a response.

Materials & experimental systems

n/a	Involved in the study
☐	☒ Antibodies
☐	☒ Eukaryotic cell lines
☒	☐ Palaeontology and archaeology
☒	☐ Animals and other organisms
☒	☐ Clinical data
☒	☐ Dual use research of concern

Methods

n/a	Involved in the study
☒	☐ ChIP-seq
☒	☐ Flow cytometry
☒	☐ MRI-based neuroimaging

Antibodies

Antibodies used

anti-ALFA single-domain antibody

Validation

The anti-ALFA sd-antibody was previously validated and shown to bind the ALFA-tag with picomolar affinity (Gotzke, H. et al. The ALFA-tag is a highly versatile tool for nanobody-based bioscience applications. Nat Commun 10, 4403, PMC6764986, doi:10.1038/s41467-019-12301-7 (2019). It is commercially available from Nanotag Biotechnologies.

Eukaryotic cell lines

Policy information about cell lines and Sex and Gender in Research

Cell line source(s)

Thermo Fisher Scientific

Authentication

Cells were received directly from vendor and were not further authenticated

Mycoplasma contamination

Mycoplasma tested every 6 months

Commonly misidentified lines
(See ICLAC register)

Not on ICLAC
